# Kainate Receptor-Mediated Depression of Glutamate Release Involves Protein Kinase A in the Cerebellum

**DOI:** 10.3390/ijms20174124

**Published:** 2019-08-23

**Authors:** Rafael Falcón-Moya, Pilar Losada-Ruiz, Antonio Rodríguez-Moreno

**Affiliations:** Laboratorio de Neurociencia Celular y Plasticidad, Departamento de Fisiología, Anatomía y Biología Celular, Universidad Pablo de Olavide, ES-41013 Sevilla, Spain

**Keywords:** kainate receptor, cerebellum, glutamate, protein kinase A, G-protein

## Abstract

Kainate (KA) receptors (KAR) have important modulatory roles of synaptic transmission. In the cerebellum, the action mechanisms of KAR-mediated glutamatergic depression are unknown. We studied these mechanisms by recording evoked excitatory postsynaptic currents (eEPSCs) from cerebellar slices using the whole-cell configuration of the patch-clamp technique. We observed that 3 μM KA decreased the amplitude of eEPSCs and increased the number of failures at the synapses established between parallel fibers (PF) and Purkinje neurons, and the effect was antagonized by NBQX under the condition where AMPA receptors were previously blocked. The inhibition of protein kinase A (PKA) suppressed the effect of KAR activation on eEPSC, and effect was not prevented by protein kinase C inhibitors. Furthermore, in the presence of Pertussis toxin, the depression of glutamate release mediated by KAR activation was prevented, invoking the participation of a G_i/o_ protein in this modulation. Finally, the KAR-mediated depression of glutamate release was not prevented by blocking calcium-permeable KARs or by treatments that affect calcium release from intracellular stores. We conclude that KARs present at these synapses mediate an inhibition of glutamate release through a mechanism that involves the activation of G-protein and protein kinase A.

## 1. Introduction

Kainate-type glutamate receptors are well-established mediators of canonical, ionotropic postsynaptic transmission and, presynaptically, these receptors support a modulatory regulation of neurotransmitter release. In the latter regard, kainate receptors (KARs) have a non-canonical metabotropic capacity, through which they effect the control of both glutamate and GABA release (for review see [1,2,3,4,5,6,7,8,9]). At several excitatory glutamatergic synapses, the KAR-mediated modulation is found to be biphasic, such that low agonist concentrations facilitate glutamate release, as opposed to higher agonist concentrations, which inhibit neurotransmitter release (see [2,3,4,5,9,10] for reviews). How this diametrically opposite modulation is mechanistically manifest is the subject of considerable debate and investigation, as is the question of the subcellular location of KARs responsible for presynaptic modulation [2,3,4,5,6,7].

KARs are expressed in the cerebellar cortex in the axons of cerebellar granule cells that form parallel fibers (PF) and form excitatory synapses with Purkinje cells (PuC) [11]. Messenger RNA transcripts encoding different KAR subunits (GluK1, GluK2 and GluK5) have been detected in granule cells, and functional expression of KAR subtypes has been reported ([12,13,14,15]. The subunits GluK1 and GluK2 have been detected on parallel fibers [15]. Biophysical studies with single-channel recordings have shown GluK1 activity [16], suggesting these KARs are Ca^2+^-permeable. A biphasic action of KARs, activated by the agonist domoate, has been shown previously at the PF-PuC synapse, with low agonist concentrations facilitating synaptic transmission and higher concentrations depressing synaptic transmission [17]. Recently we determined the mechanism by which the activation of KAR mediated the facilitation of glutamate release found at these synapses involving a calcium-calmodulin-adenylate cyclase (AC)-protein kinase A (PKA) involvement pathway [18]. Here, we have determined the mechanism underpinning the depressant effect of KA in cerebellar slices at synapses between granule cell terminals and PuC.

We found that the KAR-mediated depression of glutamate release has an obligatory dependency on G protein function and cAMP-mediated PKA activity at PF-PuC synapses in the cerebellum.

## 2. Results

### 2.1. The Activation of Kainate Receptors by 3 μM KA Produces A Decrease in the Amplitude of NMDA Receptor-Mediated Postsynaptic Currents at PF-PuC Synapses

**Figure 1:**
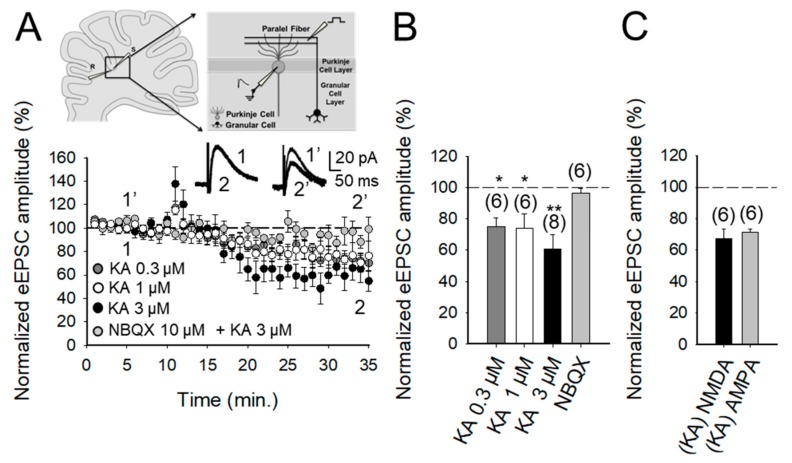
Kainate (KA) reduces the evoked excitatory postsynaptic current (eEPSC) amplitude at parallel fibers-Purkinje cell (PF-PuC) synapses of the cerebellum. (**A**) The picture shows the experimental setup. The graph shows the time course of the KA (0.3, 1 and 3 μM) effect on eEPSC amplitude in the absence (dark grey, white and black symbols) and presence of NBQX for KA3 μM (grey). The inset shows traces before and after KA 3 μM treatment in the absence (1, 2) and in the presence of 10 μM NBQX (1′, 2′). (**B**) Quantification of modulation and dose dependency of the KA effect on eEPSC amplitude in the absence and presence of NBQX. (**C**) Effect of KA (3 μM) on NMDA and AMPA receptor-mediated currents. Note that the effect of KA on these currents is indistinguishable. The number of slices (from two to three mice) is indicated in parentheses at the top of each bar. Results are expressed as the means ± SEM (* *p* < 0.05, ** *p* < 0.01, Student’s *t*-test).

Following the observation that glutamatergic transmission at PF-PuC synapses of juvenile rat pups is modulated by KARs in a biphasic manner [17], as is also the case in the hippocampus [2,3,4,5], we established the parallel fiber-Purkinje (PF-PuC) synapse paradigm in slices from early adult mouse cerebellum to study the mechanisms involved in glutamate release modulation mediated by KAR-activation. The experimental paradigm we used was the stimulation of parallel fiber axons while measuring NMDA receptor-mediated eEPSCs in PuCs, by obtaining whole-cell patch clamp recordings, with the membrane potential held at +40 mV. Recordings were made in the presence of 30 μM GYKI53655, in order to obviate AMPA receptor activation, as well as the presence of 10 μM bicuculline, to antagonize GABA_A_ receptors (in the presence of GYKI53655, AMPA currents were completely blocked from 80 ± 7 pA in control to 3 ± 2 pA in the presence of 30 μM GYKI53655, *n* = 6). In the presence of bicuculline, GABA_A_ currents were completely blocked (from 130 ± 12 pA in control to 4 ± 3 pA in the presence of 10 μM bicuculline, *n* = 6). In the presence of GYKI53655 and bicuculline, the addition of 50 μM D-AP5 completely blocked the remaining current indicating that was an NMDA current (from 60 ± 8 pA in control to 4 ± 3 pA in the presence of 50 μM D-AP5, *n* = 6). In our experiments, young adult cerebellar synapses showed detectable depression (after a transient increase) of NMDA receptor-mediated eEPSC amplitudes when 3 μM KA was applied (61 ± 7%, *n* = 8, Figure 1A,B), with 0.3 and 1 μM agonist concentrations having smaller effects (75 ± 6%, *n* = 6, 73 ± 9%, *n* = 6, respectively; transient increases in eEPSC amplitudes to: 117 ± 5%, *n* = 6 for KA 0.3 μM; 122 ± 6%, *n* = 6 for KA 1 μM and 138 ± 13%, *n* = 8, for KA 3 μM). To analyze the mechanistic details of the KAR-mediated depression of glutamatergic transmission, we hereafter utilized 3 μM KA in subsequent electrophysiological experiments as 3 μM KA produced the maximum level of depression observable. To determine whether the effect of KA recorded from Purkinje neurons in slices was mediated by the activation of KARs, analogous to that observed in other brain regions, such as the hippocampus and cortex [18,19,20,21,22,23,24,25,26,27,28,29,30,31,32,33,34], we performed experiments in the presence of NBQX. We showed that the 3 μM KA biphasic effect on the eEPSC amplitude was abolished in the presence of 10 μM NBQX (95 ± 3%, *n* = 6, Figure 1A). In these experiments, because AMPA receptors were previously antagonized in the presence of the selective blocker GYKI53655 in the bath, the observation of full antagonism by NBQX invoked the modulation to be due to KARs specifically. Further, in line with the notion that the depression (and the facilitation) of synaptic transmission observed was exclusively contingent on KAR activation.

In our studies, we confirmed a presynaptic locus of action by using several approaches. First, we performed paired-pulse recordings and measured the paired-pulse ratio (PPR; pair-pulse facilitation was observed at 40 ms pulse interval). PPR was 1.9 ± 0.4 (*n* = 8) under control/baseline conditions. After KA treatment, PPR increased to 2.2 ± 0.2 (*n* = 8), implying an effect on release probability [35], thereby corroborating the presynaptic origin of the KA receptor-mediated regulation. Second, we determined the proportion of synaptic failures in the presence of KA. Under control conditions, the synaptic failure rate was 19 ± 4%, *n* = 5. Following the application of KA, the failure rate was measurably increased (to 37 ± 8%, *n* = 6), again indicating a presynaptic locus of KA action. Finally, we compared the KA-mediated modulation of NMDA receptor-mediated eEPSCs (with GYKI53655 present) and AMPA receptor-mediated eEPSCs recorded at −70 mV (without GYKI53655, but with D-AP5 and bicuculline, to respectively block NMDA and GABA_A_ receptors). KA mediated a comparable decreased in the NMDA receptor-mediated eEPSCs (67 ± 5%, *n* = 6) and the AMPA receptor-mediated eEPSCs (71 ± 2%, *n* = 5, Figure 1C). This congruent depression of NMDA and AMPA receptor-mediated eEPSC amplitudes intimates that KA-modulation occurs presynaptically, through decreased glutamate release. Together, the preceding evaluation reliably shows a presynaptic locus of action of KA at the PF-PuC synapses under investigation. 

### 2.2. KAR-Mediated Depression of Glutamatergic Transmission at PF-PuC Is Contingent on cAMP-Dependent Signaling

**Figure 2:**
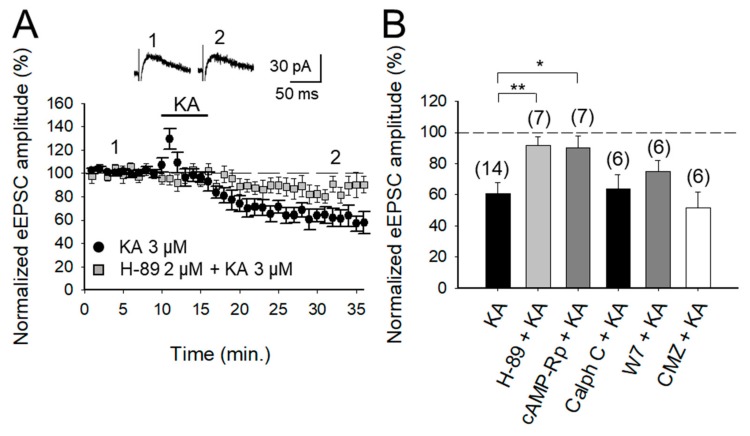
Adenylyl cyclase (AC) and downstream protein kinase A (PKA) underly the KA-mediated depression of glutamate release in PF-PuC synapses. (**A**) Time-course of the effect of KA on eEPSC amplitude in control and H-89 treated slices. Inset shows representative traces showing that KA (3 μM) does not decrease the amplitude of the eEPSCs in H-89 treated slices. (**B**) Summary of results. Blockade of PKA by H-89 (2 μM) or cAMP-Rp (100 μM) prevented the depressive action of KA. Blockade of protein kinase C (PKC) with calphostin C (1 μM) had no effect on the KAR-mediated decrease of the eEPSC amplitude (when compared to the first bar, KA). Depression similar to non-treated slices was observed on eEPSC amplitude in slices treated with 25 μM W-7 or 1 μM CMZ. The number of slices (from two to three mice) is indicated in parentheses at the top of each bar. Results are expressed as means ± SEM (* *p* < 0.05; ** *p* < 0.01, ANOVA test).

With the selectivity of the action of KA and the locus of action verified, in subsequent experiments we examined the second messenger system that mediates the depression of eEPSCs. First, we tested whether PKA was involved in the decreased eEPSCs, by inhibiting either the modulatory or catalytic activity of the kinase, by using the cAMP-Rp or H-89. With the addition of 2 μM H-89 or 100 μM cAMP-Rp, the depression of the eEPSC amplitude by 3 μM KA was prevented (93 ± 5%, *n* = 7 after H-89 and 91 ± 8%, *n* = 7 after cAMP-Rp, vs. KA 3 μM, 60 ± 7%, after a transient increase of eEPSCs to 126 ± 5% of baseline amplitude, *n* = 14 Figure 2A,B). The data together point to PKA playing an obligatory part in the observed KAR-mediated modulation of PF-PuC cerebellar glutamatergic transmission. However, given that in other slice preparations, protein kinase C (PKC) has also been implicated in aspects of the KAR-mediated modulation [2,3,9], we examined whether this kinase plays a role in the modulation of the PF-PuC cerebellar synapse by KA. In slice experiments using calphostin C (1 μM) to specifically inhibit PKC, no prevention of KAR-mediated depression was found (64 ± 9%, *n* = 6, Figure 2B), therefore obviating an involvement of PKC in the modulation observed.

The mechanisms mediating KAR-mediated facilitation of glutamate release involve calcium-calmodulin activation of AC [18]. We wanted to check if for the observed depression of the transmission the same mechanism operates. We examined this at the cerebellar PF-PuC synapse by treating slices with the calmodulin antagonist, W-7, before recording eEPSCs. With W-7 (25 μM) present, KA (3 μM)-mediated depression was not affected (75 ± 7%, *n* = 6, with W-7 vs. 60 ± 7%, *n* = 8 without W7, in interleaved slices; Figure 2B). We additionally performed experiments in the presence of calmidazolium (CMZ, 1 μM), an alternative calmodulin antagonist. As with W-7, in presence of CMZ, KA (3 μM)-mediated depression of synaptic transmission was not abrogated (52 ± 10, *n* = 6, Figure 2B). These data show that a presynaptic Ca^2+^-calmodulin complex is not necessary for KAR-mediated depression of glutamate release at PF-PuC synapses.

### 2.3. KAR-Mediated Depression of Glutamate Release Requires G Protein at PF-PuC Synapses

**Figure 3:**
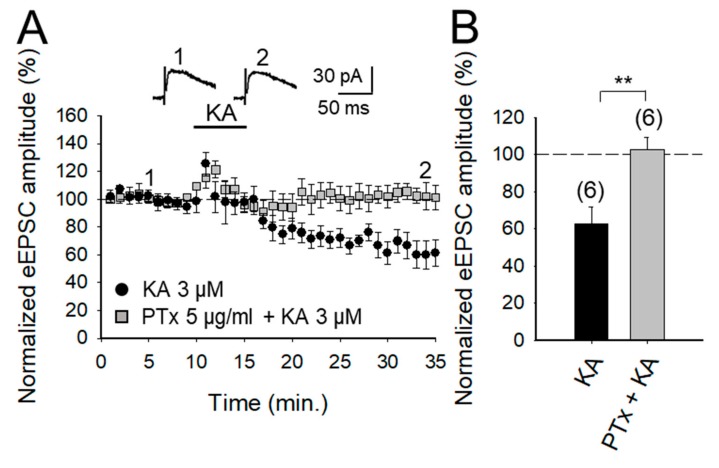
KAR-mediated depression of glutamate release is prevented in slices treated with pertussis toxin. (**A**) Time-course of the effect of KA on eEPSC amplitude in control slices and in slices treated with pertussis toxin. Inset shows representative traces showing that KA (3 μM) does not affect the amplitude of the eEPSCs in pertussis toxin-treated slices. (**B**) Summary of results. The number of slices (from two to three mice) is indicated in parentheses at the top of each bar. Results are expressed as means ± SEM (** *p* < 0.01, Student’s *t*-test).

G_i/o_ proteins have been reported to be involved in KAR signaling previously since the discovery of a role in KAR-mediated modulation of GABA release in the hippocampus (see [9] for review). Additionally, a role of G protein in KAR-mediated modulation of glutamate release has also been described in the hippocampus [36] and in the amygdala [37]. To determine whether a similar mechanism operates in the cerebellum to modulate glutamate release, we examined here the effect of KA on slices treated with Pertussis toxin (PTx, 5 μg/mL). We found that the inhibitory effect of KA was indeed suppressed by PTx, implying selective G-protein involvement in inhibitory modulation by KAR in the preparation under study (102 ± 6%, *n* = 6; vs. control 62 ± 9%, after a transient increase of eEPSC to 131 ± 9% of baseline amplitude, *n* = 6, Figure 3A,B).

### 2.4. The Depression of Glutamate Release at PF-PuC Synapses is not Mediated by Calcium-Permeable KAR and does not Require Calcium Release from Intracellular Stores

**Figure 4:**
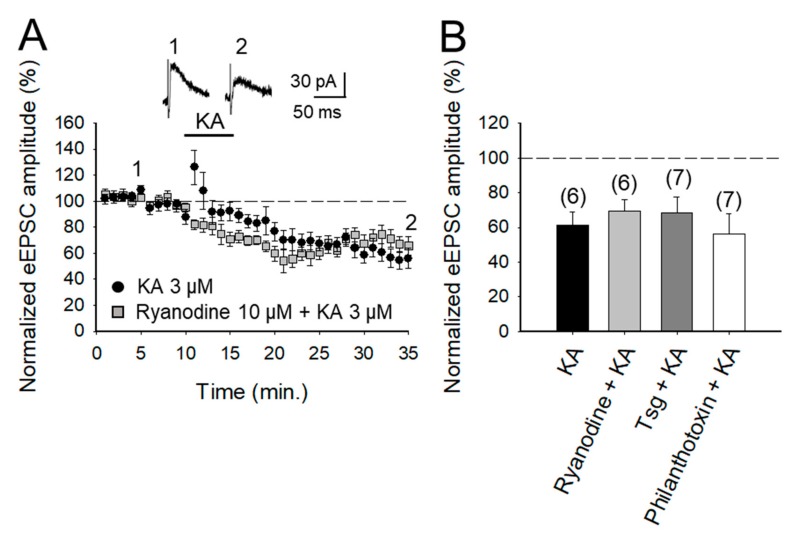
KAR-mediated depression of glutamate release does not require changes in Ca^2+^ in the cytosol at the PF-PuC synapses. (**A**) Time-course of KA (3 μM) effect on eEPSC amplitude under the control condition (circles) and in slices treated with Ryanodine (squares). (**B**) Quantification of results for slices treated with ryanodine, thapsigargin and Philanthotoxin. The decrease of eEPSC amplitudes induced by KA is not prevented in any case. The number of slices (from two to three mice) is indicated in parentheses at the top of each bar. Results are expressed as means ± SEM (Student’s *t*-test and ANOVA).

The role of Ca^2+^ in mediating KAR-mediated synaptic transmission has been subject of debate and controversy, for instance, at the hippocampal mossy fiber-CA3 (MF-CA3) synapses. Some studies suggest that permeation of Ca^2+^ through KARs and subsequent Ca^2+^-induced Ca^2+^ release from intracellular stores is obligatory for short-term and long-term plasticity at MF-CA3 synapses [21,30,38]. Others have registered no effect of KA on cytosolic [Ca^2+^] [39] and yet others advocate that a decrease in Ca^2+^ concentration underpins the modulation due to KAR activation [40,41]. To examine the requirement of Ca^2+^ at these cerebellar synapses to depress glutamate release, we investigated first the effect of ryanodine, which selectively inhibits Ca^2+^-induced Ca^2+^ release [42], to elucidate whether this underpins KAR-mediated depression. Ryanodine (10 μM) treatment had no effect on the KAR-mediated depression of transmission at these PF-PuC synapses (69 ± 9%, *n* = 7, with ryanodine vs. 62 ± 7%, *n* = 6, without ryanodine, in interleaved slices after a transient increase of eEPSCs to 124 ± 9% of baseline amplitude; Figure 4A,B). Second, to establish whether the activation of KAR causes a Ca^2+^ signal that requires amplification by mobilization of intracellular Ca^2+^ storage, we examined the effect of KA after depletion of intracellular Ca^2+^ stores, using thapsigargin to inhibit the accumulation of Ca^2+^ in the SERCA pump. Treatment with thapsigargin (2 μM) did not affect KAR-mediated depression (68 ± 9%, *n* = 6, with thapsigargin vs. 62 ± 7%, *n* = 6, without thapsigargin, in slices interspersed, Figure 4B). Finally, to corroborate the previous results and rule out that the presence of permeating Ca^2+^ KAR plays some role in the KA-dependent mechanism in these cerebellar synapses, we investigated the effect of KA on the eEPSC amplitudes in the presence of philanthotoxin, a toxin shown to block unedited, Ca^2+^ permeable KARs [30,43]. After treatment of slices with 3 μM philanthotoxin, the synaptic depression mediated by 3 μM KA was not prevented (to 56 ± 11% of initial amplitude, *n* = 7 vs. 62 ± 7%, *n* = 6 observed in interleaved slices, Figure 3B). All of these compounds were acting at the concentrations used, and while they did not block depression, they blocked the transient increase in eEPSCs observed in control slices: 84 ± 6%, *n* = 6 in ryanodine, 76 ± 6%, *n* = 7 in thapsigargin, and 77 ± 6%, *n* = 7 in philantotoxin vs. 62 ± 7%, *n* = 6 transient increase observed in control interleaved slices). Together, these results show that Ca^2+^ permeation through KAR and Ca^2+^ mobilization from internal stores has no role in the synaptic depression observed at PF-PuC synapses.

## 3. Discussion

Presynaptic KARs are well known to have a biphasic effect on the release of neurotransmitters, so that low doses of agonists produce an increase in the release of neurotransmitters, while higher concentrations produce a decrease in eEPSCs [2,3,4,5,6,7,8,9]. Although the role of the PKC pathway in some KAR actions is well recognized [2,3,4,5,6,7,8,9], some authors have found that KAR activities are not adequately ionotropic and are not mediated by protein kinases [44] or are mediated by PKA in the hippocampus [26,36,45,46] and in the amygdala [37]. Here, we show that this mechanism involving PKA is not restricted to the hippocampus and the amygdala and extend it to the cerebellum.

The results in this study, utilizing electrophysiological experiments in cerebellar slices, show that the activation of presynaptic KARs, at PF-PuC synapses, invokes a depression of synaptic transmission/glutamate release. Analysis of this modulation suggests a mechanistic coupling of KARs to PKA activity, by the activation of a G-protein. The observed KA-mediated decrease of the eEPSCs at PF-PuC synapses is due to decreased glutamate release, which could be monitored by NMDA receptor-mediated currents (with AMPA receptors antagonized by GYKI53655), and blocked by the KAR/AMPA receptor antagonist NBQX. Under conditions where the AMPA receptor activation is obviated by GYKI53655, this therefore delineates the specific role of KARs in the depressive regulation.

In assessing synaptic regulation, it is of utmost importance to identify the subcellular location of the KAR postulated. We corroborated the presynaptic presence of KARs by electrophysiological analysis of a presynaptically manifest parameter, i.e., the PPR (pair-pulse ratio) of consecutive eEPSCs mediated by neurotransmitter release. A clear increase in the PPR of eEPSCs observed with KA application in our experiments suggested a change in release probability (by definition a presynaptic property in synaptic transmission). Secondly, we assessed the proportion of synaptic failures in response to KA application. With KA application, the failure proportion was evidently increased, supportive of a decrease in the probability of presynaptic transmitter release and corroborative of the observed depression occurring through KAR activation. Finally, and importantly, the effects of KA observed on NMDA and AMPA receptor-mediated currents were similar. Given that no such equivalence would be predicted if the observed modulation was postsynaptic, the data here are supportive of a presynaptic mode of action for KARs being activated. Altogether, three independent analyses mutually corroborate and emphasize a presynaptic locus of action of KARs functioning at PF-PuC synapses. It remains to be elucidated whether the presynaptic regulation by KA at PF-PuCs reports the activity of KARs subcellularly localized at the nerve terminal/axonal or somatodendritic compartments. The technically challenging paradigms needed to address this question are beyond the scope of the present paper. However, to directly elucidate the presynaptic compartmentalization of KARs, future work necessitates: (i) high-resolution immunolocalization (immunogold-based) of the receptor (contingent on the availability of high affinity antibodies with appropriate KAR subunit-specificity); (ii) targeted blockade of KARs using caged-antagonists (contingent on the pending development of reagents) and (iii) paired recordings (see NMDA receptor studies), [47,48,49,50,51].

In agreement with our previous studies on the hippocampus and the amygdala [36,37] the inhibition of PKA by the cyclic nucleotide analog cAMP-Rp results in the cancellation of the depression mediated by KA of synaptic transmission/release of glutamate in the PF-PuC synapses. The congruence of the mechanism between the synapses is also highlighted in the current study, by observing the inhibition of the catalytic activity of PKA by H-89 [18,26,27,33,34].

In contrast to the mechanism mediating the increase in glutamate release, for depression, preventing the correct functioning of G protein by PTx treatment impedes KAR-mediated depression of glutamate release, showing that there is an initiation / transduction mediated by the G protein followed by cAMP signaling for the subsequent activation of PKA. In the canonical context, the KAR can mediate the entry of external Ca^2+^ through ionotropic activity [30,43,52], or Ca^2+^ can be released from intracellular stores, but in our results, a blockade of KAR permeable to Ca^2+^ by the selective inhibitor, philantotoxin, or the treatment of the slices with ryanodine, or thapsigargin did not cancel the synaptic depression mediated by KA, ruling out the entry of Ca^2+^ through KARs or the release of calcium from intracellular stress as the participants in the signaling process that mediate the observed depression of glutamate release mediated by KAR-activation. From our results, it is also clear that the KAR function is conserved at the PF-PuC synapses and is not limited to the first two postnatal weeks as previously reported [17]. KARs have a self-receptor role in developing animals, with the concentration of agonist that determines the presynaptic modulation: facilitation (at low [KA]) and depression (at high [KA]) and, therefore, supposedly determining the consolidation and stability of synapses. However, the modulation of the presynaptic function reported in this document may manifest some forms of plasticity as synaptic refinement may involve glutamate receptors and plasticity [53,54] and KARs have been shown to be involved in plasticity at the PF-PuC synapses (for a review, see [55,56,57]. Additionally, KARs have been involved in some brain alterations such as epilepsy [58] but whether this involves the cerebellum has not been determined yet. A direct relationship exists between KA injection and cerebellar ataxia. Thus, the cerebellum is an important target to study functions of KARs and their possible role causing ataxia [59,60,61,62]. Furthermore, in patients with schizophrenia, an increase in KARs containing GluK2 and GluK5 subunits is observed [63,64]. In neurodegeneration, it has been found that local application of KA in some areas of the cerebellum produces changes in different ion levels, highly increasing Ca^2+^ levels for weeks, which mediate calcification [65]. KARs have been described as producing increases in intracellular calcium [18,66] and KARs seems to signal increasing intracellular calcium without putting the cell at risk due to excitotoxicity, due to its low conductance in contrast to AMPARs. Due to the lack of knowledge on the subject, further exploration is necessary to determine the KAR role in cerebellum development and cerebellar alterations.

In conclusion, our study shows that the presynaptic activation of KAR by KA at the PF-PuC synapses produces a depression of synaptic transmission and a decrease in the amount of glutamate released. We postulate that mechanically, KAR-mediated presynaptic depression involves the activation of a G protein that would signal the activation of AC1 or AC8 to reduce cAMP levels to mediate a decrease in glutamate release and, therefore, in synaptic transmission at the PF-PuC synapses in the cerebellum.

## 4. Materials and Methods

### 4.1. Animals

The experiments were performed on 4–6-week-old C57Bl/6 male mice obtained from Harlan Laboratories (Spain). Experiments were conducted in accordance with the European Union directive for the use of laboratory animals in acute experiments and were approved by the local Ethical Committee (Junta de Andalucía and University Pablo de Olavide, Sevilla, Spain) (Session 2/12, 8 February 2012).

### 4.2. Slice Preparation

Cerebellar parasagittal acute slices were prepared as described previously [18] (Falcón-Moya et al., 2018). Briefly, after decapitation, the whole brain was removed under ice-cold buffered salt solution consisting of (in mM) 124 NaCl, 2.69 KCl, 1.25 KH_2_PO_4_, 2 MgSO_4_, 1.8 CaCl_2_, 26 NaHCO_3_, and 10 glucose (pH 7.2, 300 mOsm) and was positioned on the stage of a vibratome slicer (Leica 1000S) and cut to obtain cerebellar slices (350 μm thick) containing parallel fibers-Purkinje cells synapses. Slices were maintained continuously oxygenated for at least 1 h before use. All experiments were carried out at room temperature (22–25 °C). In total, 6–14 slices from 2–3 animals were used for each experiment.

### 4.3. Electrophysiological Recordings

Whole-cell patch-clamp recordings were obtained from Purkinje neurons. NMDA receptor-mediated evoked postsynaptic currents (eEPSCs) were recorded at +40 mV from these neurons visually identified by infrared-differential interference contrast (IR-DIC) microscopy using a 40× water immersion objective. Perfusion solution contained GYKI53655 (30 μM), to block AMPA receptors, and bicuculline (10 μM), to block GABA_A_ receptors. In experiments involving AMPA receptor-mediated currents, performed at −70 mV, no GYKI53655 was used, but D-AP5 (50 μM) was included to block NMDA receptors. To evoke eEPSCs, electrical pulses were delivered to granule cells axons (parallel fibers) using a monopolar electrode placed in the molecular layer at a frequency of 0.2 Hz. Patch electrodes were made from borosilicate glass and had a resistance of 4–7 MΩ when filled with (mM): 120 CsCl, 8 NaCl, 1 MgCl_2_, 0.2 CaCl_2_, 10 HEPES, 2 EGTA and 20 QX-314 (pH 7.2, 290 mOsm). A 40 ms paired-pulse stimulation protocol was used for pair pulse ratio (PPR) analysis. Neurons were voltage clamped, using a Multiclamp 700B amplifier (Molecular Devices, Foster City, CA, USA). Access resistance was regularly monitored during recordings, and cells were rejected if it changed >15% during the experiment. Data were filtered at 2 kHz, digitized at 10 kHz, and stored on a computer using pClamp software (Molecular Devices). Synaptic failures were identified as the lack of synaptic responses after presynaptic stimulation with the amplitude of these responses being no different from basal noise amplitude.

### 4.4. Data Analysis

Data were normalized, taking the control as 100% of the response, and are presented as means ± SEM. Signals were averaged every 12 traces. Effects of KA were measured at peak (maximum) compared to averaged 10 min baseline points. Significance was assessed at *p* < 0.05. A normality and equal variance test was performed before statistical comparisons. Statistical comparisons were made using two-tailed Student’s *t*-test for comparison of two data sets and analysis of variance (ANOVA) for comparison of multiple data sets using the Bonferroni as a *post hoc* test.

### 4.5. Compounds

Salts and general reagents were purchased from Sigma (St. Louis, MO, USA); GYKI 53655, D-AP5, NBQX, bicuculline, Rp-Br-cAMP, H-89, forskolin, philanthotoxin, ryanodine, thapsigargin, kainate, Pertussis toxin, CMZ and W-7 were obtained from Tocris (Bristol, UK).
